# Genetics of Cerebral Vasospasm

**DOI:** 10.1155/2013/291895

**Published:** 2013-04-11

**Authors:** Travis R. Ladner, Scott L. Zuckerman, J Mocco

**Affiliations:** ^1^Vanderbilt University School of Medicine, Nashville, TN 37212, USA; ^2^Department of Neurological Surgery, Vanderbilt University School of Medicine, Nashville, TN 37212, USA

## Abstract

Cerebral vasospasm (CV) is a major source of morbidity and mortality in aneurysmal subarachnoid hemorrhage (aSAH). It is thought that an inflammatory cascade initiated by extravasated blood products precipitates CV, disrupting vascular smooth muscle cell function of major cerebral arteries, leading to vasoconstriction. Mechanisms of CV and modes of therapy are an active area of research. Understanding the genetic basis of CV holds promise for the recognition and treatment for this devastating neurovascular event. In our review, we summarize the most recent research involving key areas within the genetics and vasospasm discussion: (1) *Prognostic role of genetics*—risk stratification based on gene sequencing, biomarkers, and polymorphisms; (2) *Signaling pathways*—pinpointing key inflammatory molecules responsible for downstream cellular signaling and altering these mediators to provide therapeutic benefit; and (3) *Gene therapy and gene delivery*—using viral vectors or novel protein delivery methods to overexpress protective genes in the vasospasm cascade.

## 1. Introduction

Cerebral vasospasm (CV) is the narrowing of the major cerebral arteries following aneurysmal subarachnoid hemorrhage (aSAH) and is a leading contributor to the morbidity and mortality associated with aSAH. The annual incidence of aSAH in the United States is between 21,000 and 33,000 people [1]. Of these, approximately 67% will develop vasospasm [2, 3]. In the setting of aSAH, CV has a biphasic course, with an acute and chronic phase. The acute phase typically begins 3 to 4 hours after hemorrhage, with rapid, spontaneous resolution. In contrast, the chronic phase begins 3 to 5 days later, with maximum narrowing between days 6 and 8, resolving after about 14 days [4].

CV can be diagnosed angiographically or clinically. Angiographic vasospasm refers to the observed narrowing of contrast medium in the major cerebral arteries. Radiologic modalities used to diagnose CV include computed tomography angiography (CTA), magnetic resonance angiography (MRA), and catheter angiography. Clinical vasospasm is the sequelae of neurocognitive deficits presumably as a result of a prolonged ischemic state. Both angiographic and clinical vasospasms can lead to cerebral infarction. Angiographic and clinical vasospasms appear to be distinct phenomena, with aSAH patients presenting with angiographic CV only (43% of patients), both angiographic and clinical CV (33% of patients), or none of them (24% of patients) [5].

Although there are many hypotheses on the pathogenesis of CV, it still remains a poorly understood phenomenon. In 1944, Zucker observed that lysed erythrocytes incited smooth muscle contraction of cerebral arteries in mammals [6]. A later clinical angiographic study by Ecker and Riemenschneider in 1951 found that the degree of vasospasm is directly related to the volume of subarachnoid blood observed on head CTs [7], leading to the use of the Fisher Grade in predicting vasospasm onset [8].

The inciting event of CV is likely an inflammatory response to extravasated blood products in the subarachnoid space, leading to prolonged and deregulated contraction of vascular smooth muscle cells (VSMCs) [9]. Lysed RBCs in subarachnoid space surrounding the cerebral vasculature can generate inflammatory downstream effects that result in endothelial damage and smooth muscle contraction [10]. In particular, extracorpuscular oxyhemoglobin is the potent inflammatory compound and has been shown to increase the formation of reactive oxygen species (ROS), decrease nitric oxide (NO) concentration, increase prostaglandin synthesis, and increase lipid peroxidation [9, 11–13]. Such changes in the vascular equilibrium can lead to the activation of pro-vasospasm signaling pathways and the synthesis of inflammatory gene products. This process occurs in parallel to the delayed cerebral ischemia (DCI) resulting from microthromboses and cortical spreading ischemia [14, 15].

## 2. Genetics in CV

While much work has been done to characterize the signaling pathways implicated in CV, the field still lacks a definitive explanatory model with robust predictability and therapeutic targets. Several presumptive targets have been proposed with only modest gains. For example, clazosentan, an endothelin receptor antagonist, has shown great attenuation of angiographic vasospasm in preclinical and clinical studies but no improvement in neurological outcomes [16]. In contrast, nimodipine, a calcium channel blocker, improves functional outcomes without a parallel reduction in angiographic vasospasm [17]. However, the field of genetics offers a new insight.

An active area of research is the exploration of the genetic basis for CV, which has previously been supported by population studies [18]. New advances in molecular genetics have revealed several genes whose products are presumptive mediators of CV and genetic polymorphisms that portend increased CV risk and/or poorer outcomes. Understanding the genetic mechanism of disease generation may provide insight into novel therapeutic avenues. In this review, we will summarize the most recent research in the following areas regarding genetics and CV: (1) prognostic role of genetics, (2) key signaling mediators involved, and (3) gene therapy and gene delivery.

### 2.1. Prognostic Factors

Stratifying risk based on next generation sequencing is gradually becoming integrated into medical practice [31]. In cardiovascular medicine, indications on the use of drugs such as clopidogrel, warfarin, and statins have already been made based on the patient's genotype [32–34]. Genetic screening of family members of patients diagnosed with familial hypercholesterolemia is currently recommended in the UK [35].

Genetic risk stratification for CV holds great potential. Close family members of patients with aneurysms may be screened for their own susceptibility for aneurysm formation and rupture. Genomic biomarkers may also be used to stratify SAH patients for more intensive monitoring according to vasospasm risk, as well as informing medication administration.

Genomewide and other gene association studies have identified several genes that may play a larger role in developing quick and inexpensive screening protocols in SAH. This represents an update on a genetics review by Ducruet and colleagues [36], with attention to recent discoveries reported in the literature. The following is a summary of genetic markers associated with SAH and CV, with evidence for disease mechanisms as well as implicated polymorphisms (Table 1).

#### 2.1.1. Catechol-O-methyltransferase (COMT)

Catecholamines have been implicated in the development of acute CV following SAH [37, 38]. COMT, a key enzyme in the degradation of catecholamines, has been shown to play a role in acute CV. A rat model has shown increased expression of COMT and catecholamines following SAH induction [39]. A study of 167 Chinese Han SAH patients showed that patients with the COMT-A allele, A/A genotype were more likely to develop acute CV [40]. This polymorphism may be a biomarker for predicting poor outcomes in patients with aneurysms that may later rupture.

#### 2.1.2. Endothelial Nitric Oxide Synthase (eNOS) Gene

Endothelial nitric oxide synthase (eNOS) gene, which is found on chromosome 7q35, plays an important role in CV and other cardiovascular diseases [41]. eNOS is present in the endothelium of the major cerebral arteries and produces nitric oxide (NO), a potent vasodilator. Constitutive levels of NO inhibit platelet aggregation, vascular smooth muscle proliferation, and inflammation [9, 23, 42–44]. Perivascular oxyhemoglobin released after SAH scavenges NO generated by eNOS, decreasing NO-mediated vasorelaxation and contributing to the onset of vasospasm [45–47].

Clinically, decreased levels of NO in the CSF have been reported in aSAH patients. Overexpression of the eNOS gene has been shown to be vasoprotective in humans and canines in the setting of SAH [48, 49]. Polymorphisms of the eNOS genes are linked to intracranial aneurysm formation [50, 51] and coronary vasospasm [52]. Genetic association studies have shown that eNOS 7-786 gene SNPs predispose aSAH patients for vasospasm [19–23]. However, the various findings are contradictory, showing either an effect with the T allele, C allele, or no clear association. This apparent discrepancy is likely attributable to the heterogeneity of vasospasm definition as well as the complex regulation of the eNOS gene.

The activity of eNOS is tightly regulated at many stages, including transcription, substrate availability, cofactors, protein-protein interactions, posttranslational modifications, and dimerization [59, 60]. The literature is mixed on whether eNOS activity is increased [61], decreased [62], or unchanged [63–66] following SAH. Further, it is still unclear what role SAH plays in the phosphorylation of eNOS [66].

eNOS is physiologically a homodimer but under pathological conditions decouples and forms a ferrous-dioxygen complex which has a tendency to form superoxide radicals instead of NO [67]. Recent studies by Sabri and colleagues have shown that SAH leads to increased phosphorylation of eNOS on Ser1177 and subsequent uncoupling, which decreases NO availability while simultaneously increasing the production of superoxide. Superoxide may further react with remaining NO to form peroxynitrite, which continues to deplete residual NO [61]. While it is known that simvastatin can mitigate vasospasm [63, 68], recent work suggests that simvastatin may work by recoupling eNOS to increase NO production and decrease the production of superoxide [61]. This may further explain its mechanism of attenuating vasospasm clinically and in other animal models [63, 64].

Another recent study showed that preconditioning (PC) of mice in hypoxic chambers prior to induction of SAH selectively increases the expression and activity of eNOS, increasing NO levels and reducing vasospasm [66]. These results suggest that PC-induced vasoprotection is mediated by upregulation of eNOS. In addition, decreased NO availability with no change in eNOS activity was observed in SAH, which may also support the pathologic uncoupling hypothesis.

#### 2.1.3. Haptoglobin

Haptoglobin (Hp) is a serum protein produced primarily by hepatocytes [69] that binds to free hemoglobin released by lysed erythrocytes [70]. The bound complex is taken up by macrophages through the CD163 receptor, presumably reducing extracorpuscular hemoglobin toxicity [71]. It is composed of an alpha subunit and a beta subunit, which are encoded by the haptoglobin alpha gene and haptoglobin beta gene, respectively. The gene complex is found on human chromosome 16q22 [72].

The Hp gene exists in 2 alleles in humans, and 3 genotypes are possible: Hp 1-1, Hp 2-2, or Hp 2-2. The Hp 1 product is a linear dimer, the Hp 2-1 product is a linear polymer, and the Hp 2-2 product is a cyclical polymer [73, 74]. These structural differences confer different binding affinities, with greater binding and clearing of hemoglobin in Hp 1-1 individuals and weaker binding and clearance in Hp 2-2 individuals [45, 75]. As a result, the Hp 2-2 allele is thought to be more proinflammatory than the Hp 1-1 allele, leading to increased inflammation, immune response, oxidation, and vasoconstriction [9, 47].

As such, the Hp 2-2 allele may be a mediator of CV as well. The Hp gene has been shown to be upregulated in a canine model of CV [76]. A study of 32 Fisher Grade 3 SAH patients conducted in 2006 showed that individuals with the Hp 2 allele had greater likelihood than Hp 1 individuals of developing vasospasm [24]. Clinically, the allelic distribution of Hp in Western patients roughly corresponds to the prevalence of CV in Western SAH patients [5, 77]. In recent years the Hp 2-2 mouse has been investigated as a novel animal model for CV and its treatment [78, 79]. Mice with the Hp 2-2 genotype display greater arterial narrowing and neurological deficits compared to Hp 1-1 mice following experimental SAH [47].

#### 2.1.4. Plasminogen Activator Inhibitor-1 (PAI-1)

PAI-1 is a protein encoded by the SERPINEI gene, found on the 7q21.3-q22 chromosome [80]. It is antifibrinolytic, functioning as the principal inhibitor of tissue plasminogen activator and urokinase, which themselves activate plasmin. A common polymorphism is the 4G/5G SNP on the promoter region [81]. The 4G allele has been previously associated with higher plasma concentrations of PAI-1 in the acute setting and poorer survival after trauma [82] as well as increased PAI-1 activity in myocardial infarction [83]. PAI-1 has also been investigated in the setting of SAH for a possible link to thrombosis-related DCI, thought to contribute to the neurological deficits resulting from CV [14]. A study of 126 aSAH patients in 2004 found that the presence of the 4G allele in the 4G/5G promoter SNP is associated with increased risk for cerebral ischemia and poorer 3-month GOS outcomes relative to the 5G allele [25]. However, another study of 183 aSAH patients in 2009 found no association between the PAI-1 SNP and poor outcomes on the 1-year GOS-E scale [26]. Given the multifactorial nature of vasospasm, more work must be done to characterize this apparent discrepancy.

#### 2.1.5. Apolipoprotein E (ApoE)

ApoE, a polymorphic protein encoded by the ApoE gene, is associated with plasma lipoproteins and is involved in lipid transport and metabolism in the central nervous system [84]. ApoE has been studied extensively in the literature for its significance to cerebrovascular disease. Three common alleles have been reported in humans (*ε*2, *ε*3, and *ε*4) on the 19p13 chromosome, encoding 3 different isoforms (ApoE2, ApoE3, and ApoE4) [85]. The *ε*4 allele predisposes patients to poor neurological outcomes in CV, traumatic brain injury, and ischemic stroke [27, 86–88]. However, the purported role of this allele in vasospasm following aSAH is still unclear. While this polymorphism does not appear to play a role in generating vasospasm, it seems to inhibit normal neuronal plasma membrane repair following cerebral ischemia, ostensibly worsening outcomes for the patients that do end up developing vasospasm [36].

Within the past decade the promoter region of ApoE has come under more scrutiny for its role in CV after SAH. Polymorphisms in the promoter region have previously been associated with poorer outcomes in traumatic brain injury. A study in 2003 found that carriers of the G-219T allele had more unfavorable GOS functional scores [89]. Another study reported that in e4 carriers the 491AA SNP contributed to poor outcomes in traumatic brain injury [90]. A study of 101 SAH patients in China in 2010 showed that patients carrying the −219T allele had an increased risk of CV [28]. This effect is possibly mediated through decreased transcriptional activity of ApoE, which decreases its protective effect in the setting of inflammation [28].

A recent study of ApoE knockout mice [91] observed enhanced vasoconstriction in response to endothelin-1, a vasoconstrictive compound associated with CV [92]. Administration of cilostazol was reported to decrease endothelial dysfunction in knockout mice, which likely increases eNOS phosphorylation [93]. This suggests that ApoE may play a vasoprotective role in CV and that underexpression of the gene may be overcome with medication for patients with normal eNOS expression in the setting of SAH.

#### 2.1.6. Ryanodine

Ryanodine receptors (RyRs) are a family of Ca^2+^ intracellular calcium channels that mediate the calcium-induced calcium release (CICR). There are three subtypes (RyR1, RyR2, and RyR3), which are all present in vascular smooth muscle [94, 95]. Activation of these receptors facilitates Ca^2+^ sparks, which promote vasorelaxation through hyperpolarizing VSMCs [96–98]. RyRs are involved in the regulation of cerebral artery luminal diameter [99, 100], and recent evidence indicates that RyRs may play a role in the pathogenesis of vasospasm after SAH. Koide and colleagues found a 50% reduction in Ca^2+^ spark activity coupled with a 65% reduction in RyR2 expression following induced SAH in rabbits [96]. These changes appear to come from a combination of reduced RyR2 expression as well as increased FKBP12.6 expression, a stabilizer of RyR2 channels. Clinically, polymorphisms in genes encoding RyRs are related to vasospasm onset. A 2011 study of 46 patients in Germany revealed that SAH patients who were heterozygous for the c.6178G>T polymorphism of the RyR1 gene were more likely to develop symptomatic vasospasm [29].

#### 2.1.7. Cystathionine *β*-Synthase

Cystathionine *β*-synthase (CBS) is an enzyme that converts homocysteine and serine to form cystathionine, releasing H_2_S in the process [101].  H_2_S is a vasodilator and neuromodulator and is known to function in the cerebral circulation, although the nature of its interaction warrants further study [30, 102, 103]. CBS is the predominant source of H_2_S in the brain and therefore may play a role in cerebrovascular disease. A study of 87 aSAH patients found that those with the 699CT and TT (gain of function) genotypes had increased angiographic vasospasm, but no increase in delayed cerebral ischemia [30]. Delayed cerebral ischemia was more frequent in 1080TT (decline of function) populations. This study suggests that H_2_S-mediated signaling is neuroprotective in aSAH, and this protection may not be dependent on vasoprotection.

More work must be done to characterize these prognostic genes and generate more consistent findings on their role in vasospasm. Differences in study designs, sample sizes, and vasospasm-monitoring modalities must be reconciled for more definitive explanations. However, these remain the most studied genes and may therefore play a role in future stratification of SAH patients.

### 2.2. Signaling Pathways

Inflammatory molecules generated from the breakdown of blood products following SAH incite several known cascades of cellular signaling enzymes. In particular, compounds such as endothelin-1, oxyhemoglobin, bilirubin oxidation products (BOXes), and ROS activate cytokine and cellular signaling pathways [9, 104, 105]. These can lead to the alteration of expression of CV-related genes, the mechanisms of which are still currently being investigated. Preliminary studies have shown that alteration of these pathways may provide therapeutic benefit. The following is a summary of 3 important known pathways in CV.

#### 2.2.1. Ras/MAPK

The MAPK signaling pathway is important in the generation of vasospasm [106]. It is hypothesized that spasmogens, when released from lysed blood cells surrounding vascular tissue, lead to the sequential activation of phospholipase C (PLC), inositol-1,4,5-trisphosphate (IP_3_), and diacylglycerol (DAG). This pathway mobilizes Ca^2+^ and activates protein kinase C (PKC), which together activate protein tyrosine kinase (TK). TK phosphorylates Ras to form the GTP-bound activated form of Ras, which is associated with cell cycle regulation, cell adhesion, and migration [107]. ROS, as those generated following SAH, have also been associated with Ras activation [108].

Ras has been shown to be activated in a rabbit model of SAH, peaking at day 3 [107]. GTP-bound (activated) Ras interacts with Raf-1 to phosphorylate downstream effectors such as extracellular signal-regulated kinase (ERK). As such, inhibition of the Ras-ERK pathway is associated with a reduction in vasospasm in rabbit and canine models of SAH [107, 109, 110].

MAPK is associated with vasoconstriction, impaired vasorelaxation, tissue proliferation, apoptosis, and inflammation [106, 111]. An *in vitro* model using human vascular smooth muscle cells found that inhibition of p38 MAPK resulted in decreased vasospasm and cytokine production [112]. It is hypothesized that caldesmon and calponin, which are substrates for MAPK, are associated with vasoconstriction, but the exact interaction has not yet been determined [106, 113]. These proteins are inhibitors of Ca^2+^-dependent smooth muscle contraction, and inhibition of them by MAPK may lead to sustained vascular smooth muscle contraction [106].

#### 2.2.2. JAK/STAT Signaling

The JAK/STAT signaling pathway is an important mediator of downstream cytokine and growth factor activity [114] and may be involved in the pathogenesis of CV. Cytokines such as IL-6 are known to be elevated in SAH patients and are also known activators of JAKs. JAK1 has been shown to be activated in a rat model of SAH following production of IL-6 [115]. JAK2 has also been shown to be activated in a rabbit model of SAH [116]. Activated JAKs subsequently phosphorylate STAT proteins. STAT proteins have been shown to be activated in the basilar artery in a rat model of SAH, with STAT3 expressed in the intima and media and STAT1 expressed in the adventitia [115]. While STAT3 is activated in response to cytokines, STAT1 is activated by the free radicals generated by oxyhemoglobin metabolism following SAH [117].

JAK2/STAT3 signaling is associated with the expression of the apoptotic genes bcl-2 and bcl-xL in the intima of the basilar arteries in rabbits [116]. JAK1/STAT3 signaling in rats upregulates the inflammatory COX-2 protein in the intima [115]. These early gene products may mediate the generation of vasospasm, as fibrosis of the cerebral arteries is associated with vasospasm following SAH [118]. Endothelial cell death promotes thrombosis and decreases vasodilator expression [119–121]. COX-2 products, including prostaglandins and thromboxanes, are known to lead to endothelial dysfunction through endothelial-dependent contractions [122]. Inflammatory changes in the adventitia may result in decreased vessel compliance and may contribute to the vessel stiffness observed in CV [120]. Taken together, these findings suggest that the JAK/STAT pathway may be an important mediator of vasospasm.

#### 2.2.3. Rho/Rho-Kinase

Rho proteins are small G proteins that are commonly expressed in mammals [123]. Rho-kinase, the effector of Rho, plays an important role in the cardiovascular system through its interaction with the myosin light chain (MLC) in VSMC contractions. Rho-kinase phosphorylates and inhibits myosin light chain phosphatase and therefore increases contractility [124]. Rho-kinase also phosphorylates myosin light chain directly, generating sustained contraction in a similar manner as the Ca^2+^/calmodulin-dependent MLC kinase pathway [125]. Rho-kinase has been shown to be involved in the pathogenesis of both coronary and cerebral vasospasm [124, 126–131]. Oxyhemoglobin from SAH activates Rho/Rho-kinase signaling [127]. In addition, the Rho/Rho-kinase pathway decreases NO production through the production of cyclophilin A (CyPA), which decreases eNOS expression [132]. CyPA itself stimulates ERK1/2, Akt, and JAK in VSMCs, which contributes to increased ROS production [133, 134]. Rho-kinase is also known to play a role in vascular smooth muscle through increasing vascular smooth muscle proliferation, ROS production, inflammation, and endothelial damage [123, 134]. Fasudil, an inhibitor of Rho-kinase, has shown some benefit in treating vasospasm in SAH patients [135].

### 2.3. Gene Therapy and Delivery

We summarize the current status of gene therapy in CV (Table 2). Within the past decade viral vector-mediated gene therapy has been explored in the context of vasospasm and other vascular diseases [136, 137]. In a proof-of-concept experiment reported in 1997, Muhonen and colleagues demonstrated that *β*-galactosidase could be transferred to cerebral blood vessels and surrounding tissue during vasospasm using a virus vector [53]. In 2002 Ono and colleagues showed that the HMOX1 gene can be transferred to the rat basilar artery adventitia through adenovirus using transcisternal injection. Overexpression of heme oxygenase-1 attenuated vasospasm in this model. This was associated with increased heme oxygenase-1 mRNA and activity, with increased basilar artery diameter and CBF [55, 138]. In the last decade this method has shown efficacy in preclinical SAH models using genes such as calcitonin gene-related peptide (CGRP) [54], eNOS [49], and superoxide dismutase [56]. There are no published clinical models of such therapy to date in CV. However, overexpression of the SERCA2a gene by viral transfer has shown success in improving outcomes in heart failure patients [139–141]. Intramuscular injection of VEGF-carrying adenovirus has improved peripheral artery occlusive disease and coronary artery disease in clinical trials [137, 142].

#### 2.3.1. Challenges

Despite the promise gene therapy holds, there are several challenges to the translation of preclinical protocols to humans in the setting of CV [136]. The route of administration is typically through the cisterna magna, which requires more invasive procedures in critically ill patients; however, external ventricular drains may provide appropriate CSF access. It is difficult to ensure adequate and accurate tissue distribution, especially to the endothelium. In addition, humans have increased body weight relative to small laboratory animals. Therefore, greater loads of gene-carrying viruses will be required. As CV is a polygenic disorder, single gene therapy alone may not be sufficient. It may be more difficult to express genes within deeper layers of blood vessels with perivascular administration. Perhaps the development of endovascular administration with vectors with enhanced transfer efficiency may be a solution. There have also been problems with expressed genes remaining functional for only short periods of time, perhaps from weeks to months. However, given the relatively short time course of vasospasm, this may not matter as much.

Safety concerns such as inflammation, viral cytotoxicity, and random viral DNA integration into host cells still persist [136]. These events may be especially problematic in CV, as an increase in inflammation may exacerbate existing vasospasm, and such responses have been shown in adenovirus gene therapy [55]. Fortunately in human trials of angiogenesis gene transfer for vascular disease, no increases in tumors, retinopathy, kidney failure, or cardiovascular endpoints have been reported [142].

#### 2.3.2. Alternatives

There are other potential approaches to targeting CV-related genes which involve delivering gene products to vascular tissue. Two recent developments are summarized below.

#### 2.3.3. Arginine-Conjugated Gene Delivery

Presumptive gene products can be conjugated with 10–20 amino acid polypeptides and delivered into somatic cells [143]. This form of protein therapy can be used for therapeutic overexpression of genes in CV. In 2011 Ogawa and colleagues demonstrated that HMOX1 can be conjugated with an 11 arginine polypeptide and introduced by transcisternal injection in a rat model. They found that this method transduced the HMOX1 gene into all layers of the basilar artery and was vasoprotective in an experimental SAH model [143]. Such a method may be applicable to transduction of other vasoprotective genes previously discussed in order to mitigate CV. However, concerns such as short time course of action, the need for continuous administration, imprecise delivery, and the need for large doses may make this less practical. In addition, this study reported no neurological differences in treated rats.

#### 2.3.4. Intranasal Protein Delivery

Advances in drug delivery have made targeted therapy even more attractive. Crossing the blood-brain barrier (BBB) has been a historical challenge in neurotherapeutics. In addition, decreased cerebral blood flow (CBF) in the perivasospasm period decreases the delivery of intra-arterial administered drugs. In 2011 Ogawa and colleagues demonstrated that intranasal delivery of calcitonin gene-related protein (CGRP) can be an effective and minimally invasive way of bypassing the BBB [58]. This study, performed in a rat model of SAH, attenuated vasospasm of the basilar artery as well as neurological deficits. More work will need to be done to see if this method is amenable to other known medications and gene products.

## 3. Conclusion

Within the past decade there has been increased knowledge in the genetic basis of CV along with refinements in gene therapy. Advances in genetic technology could add genetic screening and gene delivery to the armamentarium of future providers caring for SAH patients. While further study will be required to translate the available knowledge into clinical practice, the field of genetics holds great promise for the management of cerebral vasospasm.

## Figures and Tables

**Table 1:** Summary of prognostic genes in cerebral vasospasm.

Gene	Reference	*N*	Finding
eNOS	[19]	141	T-786C promoter → clinical/angiographic and/or TCD vasospasm
eNOS	[20]	51	T-786C SNP → symptomatic/asymptomatic angiographic vasospasm
eNOS	[21]	136	No relationship
eNOS	[22]	347	T-786C SNP → angiographic vasospasm
eNOS	[23]	77	T-786C SNP → angiographic vasospasm
Hp	[24]	32	Hp 2 allele → angiographic and/or TCD vasospasm
PAI-1	[25]	126	4G allele → DCI and poor 3-month GOS
PAI-1	[26]	183	No relationship b/w allele and 1-year GOS-E
ApoE	[27]	206	ApoE4 allele → poor GOS
ApoE	[28]	101	−219T→ clinical and TCD vasospasm
RyR1	[29]	46	GT c.6178G→T → symptomatic vasospasm
CBS	[30]	87	699CT and TT → angiographic vasospasm, but no increase in delayed cerebral ischemia. 1080TT → DCI

**Table 2:** Summary of preclinical *in vivo *gene therapy experiments.

Gene	Reference	Method	Organism	Expression	Effect
*β*-galactosidase	[53]	Adenovirus	Canine	Leptomeninges, ependyma, and BA adventitia	—
CGRP	[54]	Adenovirus	Rabbit	CSF, BA adventitia, and perivascular tissue	Attenuation of vasoconstriction
HMOX	[55]	Adenovirus	Rat	HO-1 mRNA and protein in BA adventitia	Attenuation of vasoconstriction
eNOS	[49]	Adenovirus	Canine	BA adventitia	Attenuation of vasoconstriction
Superoxide dismutase	[56]	Adenovirus	Rabbit	BA adventitia	Attenuation of vasoconstriction
CGRP	[57]	Protein	Rat	—	Attenuation of vasoconstriction
HMOX	[58]	Arginine	Rat	All layers of BA	Attenuation of vasoconstriction
